# Prioritizing the Determinants of Social-health Inequality in Iran: A Multiple Attribute Decision Making Application

**DOI:** 10.5812/ircmj.12607

**Published:** 2014-04-05

**Authors:** Rouhollah Zaboli, Sogand Tourani, Seyed Hesam Seyedin, Alireza Oliaie Manesh

**Affiliations:** 1 Department of Health Services Management, School of Health, Baqiyatallah University of Medical Sciences, Tehran, IR Iran; 2 Hospital Management Research Centre, Iran University of Medical Sciences, Tehran, IR Iran; 3 Health Management and Economics Research Centre, Iran University of Medical Sciences, Tehran, IR Iran; 4 Department of Healthcare Financing and Payment, National Institution of Health Research, Tehran University of Medical Sciences, Tehran, IR Iran

**Keywords:** Social Determinants of Health, Socioeconomic Factors, Iran

## Abstract

**Background:**

One of the main challenges of healthcare systems of developing countries is health inequality. Health inequality means inequality in individuals’ ability and proper functioning, resulting in inequality in social status and living conditions, which thwarts social interventions implemented by the government.

**Objectives:**

This study aimed to determine and prioritize the social determinants of health inequality in Iran.

**Materials and Methods:**

This was a mixed method study with two phases of qualitative and quantitative research. The study population consisted of experts dealing with social determinants of health. A purposive, stratified and non-random sampling method was used. Semi-structured interviews were conducted to collect qualitative data along with a multiple attribute decision making method for the quantitative phase of the research in which the TOPSIS technique was employed for prioritization. The qualitative findings were entered into NVivo for analysis, as were the quantitative data entered into MATLAB software.

**Results:**

The results approved the suitability of the conceptual framework of social determinants of health suggested by the WHO (world health organization) for studying social determinants of health inequality; however, this framework general and theoretical rather than a guideline for practice. Thus, in this study, 15 themes and 31 sub-themes were determined as social determinants of social health inequality in Iran. Based on the findings of the quantitative phase of our research, socioeconomic status, living facilities such as housing, and social integrity had the greatest effect on decreasing health inequality.

**Conclusions:**

A major part of the inequality in health distribution is avoidable because they are mostly caused by adjustable factors like economic conditions, educational conditions, employment, living facilities, etc. As in the majority of developing countries the living and health conditions are the same as Iran, the findings of this study may be applicable for other developing countries.

## 1. Background

Health is a special goods and its proper distribution should be the main concern of policy makers (1, 2). Inequality in health causes inequality in individuals’ ability and proper functioning. Such inequality will systematically cause unequal social status and living conditions resulting in the failure of the government’s social interventions (3). The concept of equality is a target set for health policies and the concept of health is important and valuable to every individual (4). The international framework for human rights emphasizes on the approach for health justice through concentrating on the social determinants of health. This framework is based on the universal declaration of human rights according to which each individual and their family are entitled to standard living conditions in terms of health and well-being including food, housing, healthcare and social services (5, 6).

Today, there is strong scientific evidence for the enormous effect of social indicators such as social class, social deprivation, urban marginality, stress, early childhood development, unemployment, social support, addiction, food, transportation, urbanization, immigration, and globalization, on health (7-10). If the social determinants of health in a society do not receive proper attention, healthcare provision will not significantly enhance individuals’ health. As a result, the paradigm that suggests healthcare alone can enhance living conditions has been disparaged (11, 12). Scientifically, eradication of healthcare inequality mandates elucidating the relationship between social factors and their effect on health (13). According to the Commission of Social Determinants of Health, health inequality is a toxic combination of social policies, plans, unjust economical arrangements, and wrong policy making (14-16). One of the models used to study inequality in health was first introduced by the Netherlands. This model suggests the following measures should be taken regarding policy making for social determinants of health policies: reducing the unequal distribution of socioeconomic factors or structural variables, effective control of intermediary determinants of socioeconomic factors, control of reverse effects of health on socioeconomic status and finally concerns about curative healthcare services in curative healthcare systems (17). Social factors determine health-relevant behaviors and health consequences. Effective health interventions require community-oriented approaches as the determinants of social health approach (18). From the early 1990s to 2000, social determinants of health were considered as the main concern of countries, but evidence shows that the social measures taken by these countries, particularly developing countries, to decrease the inequality and promote health justice were not successful (19-22). In order to decrease health inequality, countries have taken different actions. The British health inequality decrease plan based on social factors, the Swedish determinants-oriented national public health strategy, free market economy, Neo-liberalism and community-oriented medicine in the developing countries of the Latin Americas, East Mediterranean, Asian and African regions, all looked for the social roots of illnesses (10, 23-27).

Commission on Social Determinants of Health was founded in 2003 to study health justice. Lee (2005) believed that countries’ health interventions aimed at tackling diseases and saving lives failed and were not able to decrease inequality and injustice. The 2008 report of this commission encouraged action against health inequality in different countries to fill the gaps between socioeconomic and political factors through research about and identification of social determinants of health (28-31). Commitment to health justice requires a health-gradients approach in which not only the cause of inequality is studied but also the differences in lifestyles and living standards among different socioeconomic groups are accounted (32-35). Despite the 20th century improvements in the global general health, health inequality has increased and evidence shows that in order to prevent health inequality, social determinants of health should be attended to. Countries should attempt to reduce health inequality by paying attention to social determinants of health (36-39). The coordination of the health sector with other relevant sectors provides an optimal opportunity but there are numerous challenges and inconveniences. Iran’s health care system regarding demographic factors, health status indicators such as responsiveness and health equity is not appropriate when compared to developed countries and some Middle East countries. Despite the improvement in health status during the past 30 years, health inequalities have produced various problems in our country. Resource decentralization, serious problems of efficiency, quality of care, lack of access to minimum basic needs, and other social determinants; social determinants of health approach is needed. Based on the conceptual framework of social determinants of health inequality, this research used the multiple attribute decision-making method by considering the local development conditions of the country and priorities for policy making.

**Figure 1.**
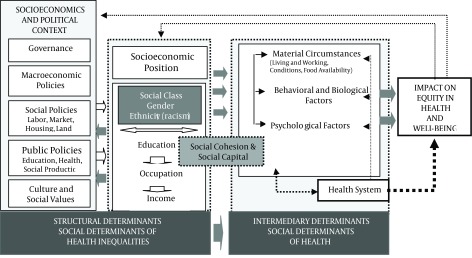
Final Form of the CSDH Conceptual Framework (40)

## 2. Objectives

This study aimed to determine and prioritize the social determinants of health inequality in Iran.

## 3. Material and Methods

This study was a mixed method research conducted in two phases:

### 3.1. Phase 1: Qualitative Phase

In this phase, the study population consisted of experts in social determinants of health. The inclusion criteria were as follows: being an expert with a minimum of three years experience, holding a PhD degree in health management & policy, clinical and social sciences and related specialties. Purposive non-random stratified sampling was used. Tashakkori and Teddlie reported that with this sampling method, the cases were purposively and non-randomly selected (41). Maximum variation sampling was used for data collection. It was estimated that the sample size of this study would be 40 experts. We also used semi-structured interviews to collect qualitative data. This guideline was made of a number of main questions regarding social determinants of health. We did not carry out the literature review with a setting of social determinants because our conceptual framework was based on the World Health Organization; this is indicated in Figure 1. Before interviewing, a pilot interview was conducted on a small scale. Interviews were continued to the point of data saturation. The number of participants in this study was 24 experts. Data were collected from experts during the period between December 2012 and April 2013 in the city of Tehran, Iran.

 After the interviews were over, they were transcribed and a version of the transcription was sent to the interviewee for confirmation. Framework analysis was used next and then thematic analysis was implemented with Nvivo to determine the main themes and sub-themes. According to the framework analysis these steps were conducted:

1. familiarization,
2. identification of a thematic framework,
3. indexing,
4. charting and
5. mapping and interpretation.

The coding of the manuscripts was guided by a primary coding frame derived from the conceptual framework of social determinants of health also research questions. Within this, new codes were developed, searching both for key concepts and for passages aimed at illustrating specific themes and sub-themes. The main social determinants of health inequalities were extracted in the qualitative phase while in the quantitative phase they were prioritized.

### 3.2. Phase 2: Multiple Attribute Decision Making Analysis Via TOPSIS

There are various models to rank and prioritize the measures taken by different researches; the most well known among models is the family of multiple attribute decision making which uses techniques such as the technique for order of preference by similarity to ideal solution (TOPSIS) which are widely used in a variety of science disciplines due to their practicality. The multiple attribute decision making approach was selected as the data collection method for the quantitative phase. By the TOPSIS technique, the criteria were weighted and chosen in accordance with qualitative studies and the experts’ knowledge. TOPSIS was introduced by Hong-Lee and Lei. TOPSIS was selected as one of the compensatory classic methods in multiple attribute decision making to solve the problems of prioritization based on similarity to ideal solution (42, 43). The TOPSIS method, a multiple attribute decision-making (MADM) technique, was used to perform the prioritization in this study. The SAW method is exploited to identify the weights of each factor. The experts could use the following nine-points for expressing the intensity of the preference for one criterion versus another:

1 = Equal importance or preference.

3 = Moderate importance or preference of one over another.

5 = Strong or essential importance or preference.

7 = Very strong or demonstrated importance or preference.

9 = Extreme importance or preference.

The TOPSIS technique consists of the following steps (43):

1. Compute the normalized decision matrix. The normalized value r_ij_ is calculated as (Equation 1):
  Equation 1. $$r_{\mathrm{ij}}=\frac{f_{ij}}{\sqrt{\sum_{j}^{J}{f^{2}}_{\mathrm{ij}}}}$$
  J = 1, J; i = 1, n
2. Calculate the weighted normalized decision matrix. The weighted normalized value vij is calculated as:
  V_ij_ = w_i_r_ij_
  j=1, J; I = 1, n
  Where w_i_ is the weight of the ith attribute or criterion, and (Equation 2)
  Equation 2. $$\sum_{i=1}^{n}w_{i}=1$$
3. Determine the ideal and negative-ideal solution (Equations 3 and 4).
**Equation 3.**
  A^+ ^={V_i_^+^ ,… V_n_^+^}={(max⁡V_ij_ | i ∈ I^"^ ),(min V_ij_ | i ∈ I”)}
**Equation 4.**
  A^- ^={V_i_^-^ ,… V_n_^-^}= {(min Vij | i ∈ I^´^),(max V_ij_ | i ∈ I”)}
  Where I´ is associated with advantage criteria and I” is associated with cost criteria.
4. Calculate the separation measures, using the n-dimensional Euclidean distance. The separation of each alternative from the ideal solution is given as (Equation 5):
  Equation 5. $$D_{j}^{+}=\sqrt{\sum_{i=1}^{n}(v_{\mathrm{ij}}}-v_{i}^{+})²$$
  j = 1, J
  Similarly, the separation from the negative-ideal solution is given as (Equation 6):
  Equation 6. $$D_{j}^{-}=\sqrt{\sum_{i=1}^{n}(v_{\mathrm{ij}}}-v_{i}^{-})²$$
  j = 1, J
5. Calculate the relative closeness to the ideal solution. The relative closeness of the alternative aj with respect to A* is defined as (Equation 7):
  Equation 7. $$C_{j}^{+}=\frac{D_{j}^{-}}{D_{j}^{+}+D_{j}^{-}}$$
  j = 1, J
6. Rank the preference order.

The whole process was performed by the MATLAB software.

## 4. Results

In this study, 50% of interviewees were male, eight of the 24 interviewed had studied health management and policy, and seven were clinicians, three of the 24 interviewed had studied social sciences and eight had studied a related specialty. 75% of the experts (18 of the 24) were found to hold 15 years of experience or above. We found that the conceptual framework of social determinants of health presented by WHO was a proper model for studies on the aspects of social determinants of health and health inequality. Also, it was proved to be more of a general model and more theoretical than practical. In this study, 15 themes and 43 subthemes emerged as the social determinants of health and inequality in Iran. As mentioned earlier, socioeconomic position, living facilities such as housing and social integrity have the strongest influence on decreasing health inequality according to expert opinion in Iran (Table 1). 

**Table 1.** Basic Characteristics of Interviewees ^a^

Name	Sex	Subject Area Studied	Work Experience
**Expert #1**	male	health management & policy (HMP)	< 15 years
**Expert #2**	female	clinical (C)	> 15 years
**Expert #3**	female	other specialty (OS)	< 15 years
**Expert #4**	female	health management & policy	< 15 years
**Expert #5**	female	clinical	< 15 years
**Expert #6**	male	clinical	< 15 years
**Expert #7**	male	health management & policy	< 15 years
**Expert #8**	female	clinical	> 15 years
**Expert #9**	male	other specialty	< 15 years
**Expert #10**	female	other specialty	< 15 years
**Expert #11**	female	clinical	< 15 years
**Expert #12**	male	other specialty	< 15 years
**Expert #13**	female	clinical	> 15 years
**Expert #14**	male	clinical	< 15 years
**Expert #15**	male	other specialty	>15 years
**Expert #16**	male	health management & policy	< 15 years
**Expert #17**	female	social science (SS)	< 15 years
**Expert #18**	male	social science	> 15 years
**Expert #19**	male	social science	> 15 years
**Expert #20**	male	other specialty	< 15 years
**Expert #21**	female	health management & policy	< 15 years
**Expert #22**	male	health management & policy	< 15 years
**Expert #23**	male	other specialty	< 15 years
**Expert #24**	male	other specialty	< 15 years

^a^ Totals: 14 male; 14 female; HMP = 6; C = 7; SS = 3; OS = 8; < 15 years= 18; > 15 years = 6.

**Table 2.** Social Determinants of Health Inequality in Iran Based on Expert Opinion

Themes	Sub-themes
**Disaster and crisis**	vulnerable group; road accidents
**Socioeconomic position**	income; social class
**Public policies**	health policy; organizational commitment; multi disciplinary participation
**Material circumstances**	food availability; living conditions and facilities; nutrition of target populations
**Education**	health literacy; education levels; people empowerments and capability
**Social policies**	labor and employment; marginalization and immigration; housing
**Culture and religious**	religious principles and faith; social and individual behavior
**Life style**	sexual behavior; life skills; health skills and drug abuse; risk factors
**Macroeconomic policies **	public and private policies; health policy; out-of pocket
**Early childhood development**	micronutrient deficiency; childhood care
**Social protection**	comprehensive social security; insurance
**Health care system**	phc; financial; physical and technological access; media access
**Behavioral and biological factor**	cultural factor; rudeness; environment
**Social cohesion and social capital**	ngos; social capital; social network; multi disciplinary participation
**Ethnicity **	gender; gender lens
**International factor**	international sanctions and boycotts; health diplomacy

**Table 3.** Prioritizing the Social Determinants of Health Inequality in Iran by the TOPSIS Technique

Social Determinants of Health Inequality	Separation From the positive-ideal Solution D^+^_j_	Separation From the Negative-ideal Solution D^-^_j_	TOPSIS Index C^+^_j_	Rank the Preference Order
**Socioeconomic position**	0.0283	0.0591	0.6762	1
**Social policies **	0.0332	0.0675	0.6703	2
**Social cohesion and social capital**	0.0236	0.0478	0.6694	3
**Macroeconomic policies **	0.0287	0.0513	0.6413	4
**Culture and religious **	0.0315	0.0563	0.6412	5
**Public policies **	0.0280	0.0499	0.6405	6
**Social protection**	0.0311	0.0484	0.6088	7
**Early childhood development **	0.0315	0.0458	0.5924	8
**Life style**	0.0327	0.0471	0.5902	9
**Health care system**	0.0371	0.0513	0.5803	10
**International factors**	0.0348	0.0472	0.5756	11
**Disaster and crisis**	0.0366	0.0494	0.5744	12
**Behavioral and biological factors **	0.0355	0.0437	0.5517	13
**Education**	0.0354	0.0430	0.5484	14
**Ethnicity **	0.0363	0.0412	0.5316	15

## 5. Discussion

A major part of health inequality is preventable and can be decreased because it has roots in adjustable factors. Such factors may include economic status; education levels; employment and living facilities. In fact, health justice indicates that under ideal conditions, everybody has a fair opportunity to enjoy complete physical, psychological, spiritual and social health and no one should be deprived of reaching such opportunities. Economic position is one of the most important and influential determinants of health and well-being, especially in developing countries. This determinant plays its role through influencing other factors like healthcare availability, healthy nutrition, education, and housing. However, the relationship between these two variables is bilateral and a healthy population means less poverty and greater economical growth. Therefore, all political models and governmental plans focus on promoting incomes and its better distribution; in other words, they concentrate on economic justice.

 Employment, psychological and social support, urban and rural residence, socioeconomic factors and social status, and culture are the most important determinants of health inequality in developing countries (14, 18, 44-51). Moreover, the attainment of healthcare facilities in different provinces is one of the major causes of health inequality in Iran (52).

Health inequality is a special case of difference in health, where the groups that are socially vulnerable or in permanent improper and discriminative social conditions systematically suffer from severer health conditions, and are at higher risks compared to the groups enjoying an optimal social status (19). Socioeconomic inequalities and their effects on health are one of the challenges that have recently attracted attention because improving health in afflicted societies is more difficult than helping patients in a healthy society. The majority of factors causing health inequality are distributed all over different social sectors. Therefore, it is necessary to take a multi-disciplinary approach in policy-making and to evaluate the probable effects of policies on health, particularly on the health of the most vulnerable groups of the society. However, the notion of health does not extend far beyond medical care to the policy makers of some developing countries and as a result; they are not able to understand the close relationship between health and their own duties. The health sector is held responsible for informing the policy-makers of such matters.

To investigate the main roots of factors threatening health, the causes of inequality should be recognized and then, serious measures must be taken to correct them through public cooperation (11). This research also determined social integrity as one of the most important and highly prioritized determinants of health inequality. There are social issues such as poverty, unemployment, and illiteracy in each country to different extents. It is important to know that the effect of each variable on health distribution is determined first by its partial effect and second by its effect on socioeconomic distribution; they interact and have a synergetic outcome (53). The concept of justice is among basic religious concepts. Unequal opportunities can affect the health of the economy, society and families, in addition to the social and psychological health of individuals. Unequal distribution of income, employment, education, and facilities plus social class inequalities in terms of skin color, race, and nationality can lower health indices. In this research, these factors were also identified. Regarding the social determinants of health, the majority of health provision is warranted out of the health sector. Education, housing, urban planning, and welfare are the sectors with a considerably large share in health. In order to decrease inequality, new paradigms should be developed through combination of science, practice and politics (24, 32, 54). Considering the current unequal policies, restricted resources, lack of a comprehensive approach, and lack of a health approach, the determinants were prioritized. This study had some potential limitations that may affect the results. Lack of a comprehensive understanding about the social determinants of health was found as the most important limitation.

A major part of the inequality in health distribution is avoidable because they are mostly caused by adjustable factors such as economic conditions, education conditions, employment, living facilities, etc. Finally, this study was conducted with respect to Iran while there are lessons for health organizations in developing countries specially countries in the Middle East. These findings can be considered for policy making and resource distribution.
